# Rearing Management and Its Impact on Caseous Lymphadenitis in Sheep

**DOI:** 10.3390/ani14101504

**Published:** 2024-05-18

**Authors:** Nora El Khalfaoui, Bouchra El Amiri, Jean-François Cabaraux, Mouad Chentouf, Marianne Raes, Tanguy Marcotty, Nathalie Kirschvink

**Affiliations:** 1 Namur Research Institute for Life Sciences, University of Namur, 5000 Namur, Belgium; nora.elkhalfaoui@unamur.be (N.E.K.);; 2 Animal Production Unit, Regional Center Agricultural Research of Settat, National Institute for Agricultural Research (INRA), Avenue Ennasr, P.O. Box 415, Rabat Principal, Rabat 10090, Morocco; 3 Department of Veterinary Management of Animal Resources, FARAH Centre, Faculty of Veterinary Medicine, University of Liege, 4000 Liège, Belgium; jfcabaraux@uliege.be; 4 Regional Center of Agricultural Research of Tangier, National Institute for Agricultural Research (INRA), Avenue Ennasr, P.O. Box 415, Rabat Principal, Rabat 10090, Morocco; mouad.chentouf@inra.ma

**Keywords:** caseous lymphadenitis, rearing system, risk factors, superficial form, visceral form, slaughterhouses, sheep flocks, quadruplex-PCR

## Abstract

**Simple Summary:**

Caseous lymphadenitis is one of the main infectious problems affecting sheep rearing areas worldwide. In Morocco, sheep farming constitutes an important socioeconomic activity and is considered to be a source of income for the local rural population. However, breeders are facing considerable economic losses due to caseous lymphadenitis. This negative impact is mainly due to the low prices and limited saleability of sheep with superficial abscesses, particularly for religious celebrations. In the present study, investigations were realised at the flock and slaughterhouse levels to determine the prevalence and risk factors implicated in caseous lymphadenitis. As a result, the form of rearing management adopted was found to impact the dissemination of caseous lymphadenitis. In conclusion, improvement of ventilation in sheep barns and implementation of periodic control of abscess cases, including their treatment with adequate procedures, are a must.

**Abstract:**

Two surveys were conducted to assess the prevalence and risk factors of ovine caseous lymphadenitis (CL) and explore the association between its superficial and visceral forms in Sardi sheep in Settat province, Morocco. A total of 1521 sheep, including 318 lambs < 6 months, 572 young sheep aged 6–24 months, and 631 adult sheep > 24 months, were examined from 57 flocks. Superficial CL prevalence levels were as follows: 69/318 in lambs, 292/572 in young sheep, and 114/631 in adult sheep. Additionally, 2124 sheep, including 1813 young and 311 adult sheep, were inspected in slaughterhouses ante- and post-mortem. Among all infected animals, the prevalence of visceral abscesses was notably higher in adult sheep (83%, OR = 9.8, 95% CI = 5.5–17.2) compared to young sheep (35%). Data on flock size, sex, age, body condition score, rearing methods, and hygienic measures were collected. Suspected CL cases were confirmed using quadruplex-PCR. Poor barn ventilation, lack of abscess control, and younger age were identified as key risk factors for CL. Additionally, a high percentage (89%) of animals with thoracic abscesses did not display superficial lesions. While age and abscess control have been reported in previous studies, barn ventilation appears to be a new, but important, risk factor. In conclusion, the identified risk factors in Settat province are linked to breeding management practices. Implementing regular abscess control measures and improving barn ventilation are essential for CL prevention.

## 1. Introduction

Sheep play a pivotal role as a primary source of meat and milk globally [1]. By 2022, the worldwide sheep population had reached 1.3 billion individuals [2]. In Morocco, sheep farming stands as a vital socioeconomic pursuit, constituting a primary income source for local rural communities, with an estimated sheep population of approximately 21.8 million [2]. Among these, the Sardi breed emerges as one of the most prominent indigenous varieties, boasting a population estimated at 2.5 million individuals [3]. This breed holds significant cultural and religious significance and is often preferred for ceremonial occasions. However, breeders face substantial economic losses attributable to caseous lymphadenitis (CL), a contagious disease. The adverse effects of CL are primarily attributed to the reduced market value and limited saleability of sheep exhibiting superficial abscesses, which are particularly undesirable for religious observances that mandate healthy animals. Moreover, CL detrimentally impacts leather and wool production, along with reproductive efficiency [4,5]. Additionally, contaminated carcasses and trimmed sections yield lower meat quantities, contributing to economic losses [6].

Caseous lymphadenitis primarily afflicts small ruminants, although cases have been reported in various mammalian species, including camelids, horses, and cattle [7,8]. Furthermore, owing to its zoonotic potential, CL poses risks to individuals in close contact with infected animals, such as farmers, veterinarians, and shearers [9,10,11]. Clinically, it manifests as suppurative necrotic inflammation of organs and lymph nodes, caused by the facultative intracellular bacterium *Corynebacterium pseudotuberculosis*, formerly known as *Corynebacterium ovis*, *Preisz-Nocard bacillus*, and *Preisz-Guinard bacillus* [12]. Initially isolated and described by Nocard in 1888 from a cow afflicted with ulcerative lymphangitis, CL presents in two distinct clinical forms. The superficial form targets peripheral lymph nodes, including the parotid, retropharyngeal, submandibular, popliteal, prefemoral, and prescapular lymph nodes, while the second form affects mediastinal and bronchial lymph nodes, as well as visceral organs such as the lungs, spleen, kidneys, and liver [13]. After an incubation period spanning 15 to 180 days, the infection progresses to pyogranulomatous lesions [4,14], which subsequently enlarge and encapsulate with fibrous tissue [11]. Currently, no effective treatment exists for CL, as antibiotherapies often fail due to the bacterium’s intracellular nature and the formation of fibrous capsules encasing the lesions [15,16]. Consequently, preventive measures remain the most viable option for curtailing and preventing its spread.

The risk factors associated with caseous lymphadenitis (CL) vary depending on the sheep breeding management system, which differs from region to region. For example, in Dakahlia Governorate, Egypt, housing systems lacking hygienic measures and the practice of cohabiting infected sheep with healthy ones have been identified as the primary risk factors for CL infection [17]. Similarly, semi-extensive and intensive rearing systems, along with the absence of individual animal identification and participation in animal exhibitions, have been cited as risk factors for CL in Brazil [18]. Furthermore, the age of animals and their poor body condition score have also been linked to CL prevalence in numerous countries [18,19,20,21].

In Morocco, sheep breeding practices are characterized by extensive rearing systems grounded in traditional knowledge. In Settat province, there exists an underestimation of the visceral form of CL, with many farmers believing that only the superficial form prevails. However, it is likely that the proportion of animals with internal abscesses is higher than those exhibiting superficial ones [22]. Hence, it is imperative to ascertain the levels of prevalence of both the superficial and visceral CL forms, along with their respective specific risk factors in this region, in order to devise an effective preventive strategy. Thus, the objectives of the present study are twofold: firstly, to determine the prevalence and risk factors of superficial CL at the flock level, and secondly, to investigate the association between superficial and visceral forms of CL at the slaughterhouse level in Settat province, Morocco.

## 2. Materials and Methods

### 2.1. Study Area and Rearing Systems

The present study was conducted in the Beni-Meskine area, situated within the district of El Borouj (32°30′14.3″ N–7°11′21.0″ W), located in the Casablanca–Settat region in the heart of Morocco. This region experiences an arid climate, characterized by hot summers. During August, temperatures can exceed 40 °C, while January sees lows of around 5 °C. Breeding practices in this area are delineated by two distinct systems, as elucidated by El Amiri et al. [3]. The semi-extensive system involves hoggets and ewes of all ages, managed outdoors for grazing. From January to April, these animals are relatively well-fed, with adjustments made outside this period based on their physiological states (pregnant, lactating, or maintenance), albeit without consideration of their specific nutritional requirements. Conversely, the intensive system pertains to the rams and hoggets, primarily males, designated for ceremonial sacrifice. These sheep are withdrawn from pasture and confined indoors for fattening over a duration of two to three months prior to the ceremony. Alfalfa hay and barley grains constitute the primary feed components in this scenario, supplemented occasionally with lentil straw, carob, sugar beet pods (*Jelfa*), sunflower cake, and commercial compound feed.

### 2.2. Study Design

Two complementary surveys were realised. Survey 1, targeting superficial CL, was conducted between August 2019 and February 2020 on 1521 animals belonging to 57 flocks. In each flock, a random 20% sample of the animals was examined clinically to detect superficial CL abscesses. Clinical data and information about flock management were collected. Survey 2, targeting superficial and visceral CL forms, was conducted between August and November 2021 on 2124 sheep which underwent ante- and post-mortem ex-amination with detection of superficial and visceral CL abscesses in four slaughterhouses in Settat province.

### 2.3. Data Collection

#### 2.3.1. At Farm Level

A total of 57 flocks were investigated, encompassing 1521 sheep (1062 males and 459 females) out of a total population of 7605. Flock sizes ranged from 25 to 315 heads, with a median of 105.

Information regarding flock size, rearing methods, and the implementation of hygienic measures was collected and summarized in Table 1. The rearing system employed in the two- or three-month period preceding the flock investigation was documented for each animal. Additionally, the density of animals was calculated by determining the head-to-square meter ratio within the barn’s area. Barn ventilation was categorized into four levels based on the presence of openings and their surface area: Level 1 denoted barns without a roof, providing maximum ventilation; Level 2 referred to barns with wide bilateral openings for ample ventilation; Level 3 indicated barns with wide unilateral openings for moderate ventilation; and Level 4 represented barns with poor ventilation due to limited unilateral openings. Furthermore, the presence of objects within the barns that could potentially cause injuries to sheep (such as traumatizing feeders, drinkers, fences, and thorny plants) was documented.

Information regarding hygienic measures within livestock buildings was collected, including the frequency of cleaning (ranging from twice a week to daily) and disinfecting (ranging from monthly to never). The types of disinfectants used and the practice of providing crawlspace were also noted. Additionally, the application of hygienic measures concerning CL cases was recorded, encompassing periodic abscess control, including the detection and treatment of abscesses before spontaneous rupture (classified as non-controlled, irregularly controlled, or regularly controlled), the type of treatment used for abscess cases (such as bleach, bleach combined with antibiotics, cade oil, or no treatment), the practice of isolating CL cases, and the destruction of pus.

A systematic approach was employed to investigate 20% of each flock, in which every fifth sheep undergoing clinical inspection was selected for examination. The clinical assessment encompassed several parameters: age determination via inspection of teeth (categorized as lamb: younger than 6 months, young: aged from 6 to 24 months, and adult: older than 24 months), sex determination, assessment of body condition score (ranging from 1 to 5), and detection of superficial abscesses. Age estimation of animals relied on the examination of their incisor teeth, considering the transition from temporary to permanent teeth, as previously described by EL Khalil et al. [23].

**Figure 1:**
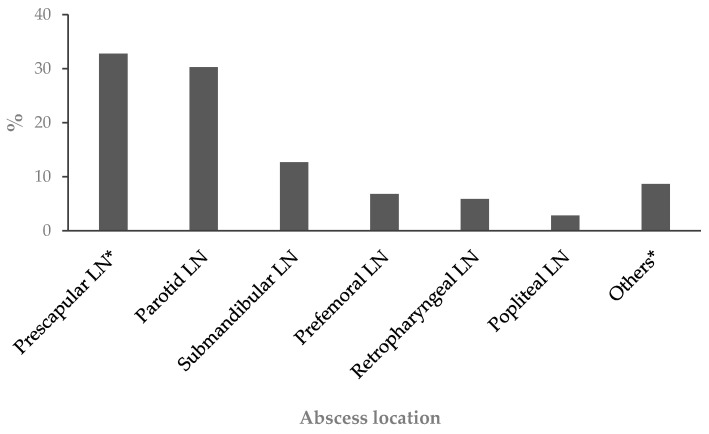
Distribution of abscess locations in superficial clinical CL cases found in sheep from the 57 visited flocks. * LN: Lymph node. Others: Popliteal lymph nodes, superficial inguinal, neck, testicles and scrotum.

The detection of abscesses was conducted through palpation of external lymph nodes, including the parotid, submandibular, retropharyngeal, prescapular, prefemoral, and popliteal nodes, as well as the testicles and scrotum in rams and the mammary gland in ewes. Additionally, subcutaneous abscesses were identified (refer to Figure 1). Information regarding the presence of single or multiple palpable abscesses, each with a diameter of at least 2 cm, along with their respective locations, was recorded.

#### 2.3.2. At Slaughterhouse Level

At the four slaughterhouses, a total of 2124 sheep underwent inspection, comprising 1813 young sheep aged between 6 to 24 months and 311 adult sheep older than 24 months. Among them, the majority were females (*n* = 2046), while 78 were males. For the survey, visits were made to the provincial abattoir of Settat twice a week, and three slaughterhouses situated in rural areas (Dar Chafii, Guisser, and Bou’Ali Nouaja) were visited weekly on different days. All selected sheep destined for slaughter underwent both ante- and post-mortem examination. Information regarding sex, age, and body condition score was recorded. Superficial abscesses were located on the body surface/skin, the head region, and superficial lymph nodes, while visceral abscesses were detected within internal lymph nodes or viscera. Descriptive data regarding all abscesses and their locations were documented. Out of the 2124 inspected animals, 423 were confirmed as CL cases, including 177 animals with exclusively visceral abscesses (116 exclusively thoracic and 61 exclusively abdominal abscesses), 225 animals with exclusively superficial abscesses, and 21 sheep with both superficial and visceral abscesses.

### 2.4. Sample Collection

Abscess material was collected from sheep exhibiting superficially enlarged abscesses across the 37 flocks. Additionally, in slaughterhouses, pus samples were obtained from all cases displaying either superficial abscesses and/or internal infected lymph nodes and viscera (totalling *n* = 497). Confirmation of *Corynebacterium pseudotuberculosis* in cases collected from slaughterhouses was established by identifying the bacterium in at least one superficial or one visceral abscess from an infected animal presenting with a CL form. Pus sampling was conducted aseptically from the periphery of drained abscesses using a single sterile swab. These samples were then transported at +4 °C in test tubes containing AMIES transport medium (Invasive Sterile Collection Swab, Yangzhou, China) for subsequent analysis. In the case of infected lymph nodes collected from slaughterhouses, they were initially immersed in a sodium hypochlorite solution to disinfect their surfaces. Subsequently, they were transported at +4 °C to the laboratory, where pus sampling was performed once the samples reached the facility.

### 2.5. Bacteriological and Molecular Diagnosis

The bacterium *C. pseudotuberculosis* was isolated using 5% defibrinated blood agar cultures and incubated at 37 °C for 48–72 h. Colonies resembling *C. pseudotuberculosis* were subjected to catalase and CAMP tests, as well as Gram staining for further analysis. Subsequently, confirmation of the colonies was carried out using quadruplex-PCR, a specific PCR method enabling both detection and typing of the *C. pseudotuberculosis* biovar, as described by Almeida et al., with minor modifications [24]. The quadruplex-PCR utilized primers targeting 16S rRNA, rpoB, and pld genes, as previously described in studies [25,26,27], along with primers targeting the nitrate reductase gene narG, as described by Almeida et al. [24]. In brief, the quadruplex PCR was conducted directly using a suspension of bacteria in a final volume of 50 µL, comprising 1× GoTaq^®^ reaction buffer (1.5 mM MgCl2) (Promega, Madison, WI, USA), 200 µM of each of the deoxynucleoside triphosphates dATP, dCTP, dGTP, and dTTP, 1.25 U of DNA Taq polymerase (Promega, Madison, WI, USA), and 0.2 µM of each primer. Amplification was carried out in a thermal cycler (PTC-100, MJ Research, Waltham, MA, USA). The amplified products were then subjected to electrophoresis in a 0.8% agarose gel prepared in 1× Tris-Acetate-EDTA buffer (40 mM Tris Base, 20 mM acetate, and 1 mM EDTA, pH 8.6), stained with Midori Green Advanced DNA Stain (NIPPON Genetics EUROPE, Duren, Germany), and visualized under UV illumination.

### 2.6. Statistical Analysis

In Survey 1, prevalence data of caseous lymphadenitis were analysed using univariable robust logistic models for all explanatory variables. The flock was considered a cluster variable to ensure the robustness of the analysis. The tested variables included sex, age categories, body score, flock size, ventilation in the building, animal density in the sheepfold, presence of traumatic equipment (drinkers, feeders, or fences), presence of thorny plants in the livestock building, isolation of diseased animals, frequency of cleaning and disinfection of livestock buildings, products used in disinfection of barns, crawlspace practice, periodic control of abscesses, treatment of abscesses, and destruction of abscess content. All of these explanatory variables, except flock size and density, were categorical. Subsequently, explanatory variables demonstrating a significant outcome were subjected to stepwise (non-robust) removal multivariable logistic regression. Two-by-two interactions between the remaining explanatory variables were tested. Finally, significant variables from the non-robust multivariable model were utilized in the final robust multivariable logistic regression.

In Survey 2, abscesses localized in the lung, mediastinal and bronchial lymph nodes, and heart were grouped together as thoracic abscesses. Similarly, abscesses in the liver and intestines were pooled as abdominal abscesses. The presence or absence of abscesses in three body regions (superficial, thoracic, and abdominal) in young and adult sheep was analysed in a robust logistic model, with animals as clusters. Additionally, the interaction between age group and abscess location was tested. The prevalence of visceral abscesses in young and adult sheep was analysed using logistic regression. Furthermore, the proportion of infected animals with superficial or visceral abscesses was evaluated in a data subset (excluding non-infected animals) through two separate logistic regressions, with the presence of superficial or visceral abscesses as response variables and age groups as explanatory variables. Additionally, the correlation between superficial and thoracic abscesses was examined using logistic regression of superficial abscesses in a data subset containing only animals with thoracic abscesses, with age classes as explanatory variables. Finally, ordered logistic regressions were separately employed for young and older animals to assess the relationship between body score and caseous lymphadenitis abscesses. Body score was considered as a response variable, while the presence of thoracic, abdominal, and superficial abscesses served as explanatory variables.

Stata 11 software (StataCorp, 2012) was utilized for statistical analyses, with a *p*-value < 0.01 considered to be significant.

## 3. Results

### 3.1. Prevalence and Risk Factors of CL Superficial Form

Clinical cases of caseous lymphadenitis (CL) were identified in the majority of the studied flocks (95%), with a mean clinical prevalence of 34.6% (ranging from 2.5% to 100%) and an overall individual prevalence of 31.1% (calculated based on the 1521 examined sheep). The univariable analysis revealed high prevalence levels associated with many factors, all of which were considered in the subsequent multivariable analysis (refer to Table 2). Only variables demonstrating significant associations with CL occurrence were included in the multivariable analysis.

The density of animals and daily cleaning of barns exhibited weak correlations with CL prevalence (*p* = 0.028) and were consequently excluded from the subsequent multivariable model.

**Figure 2:**
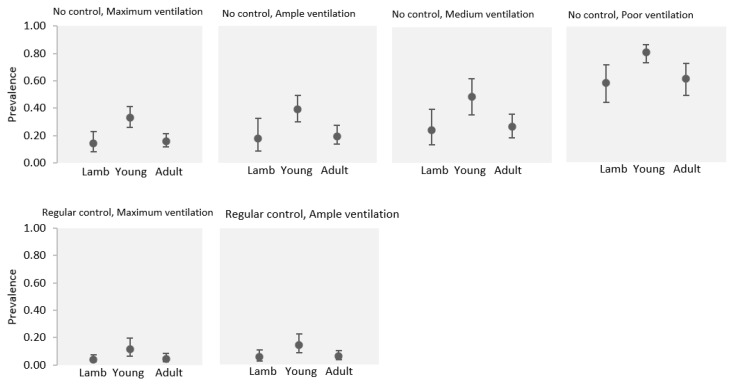
Clinical prevalence of superficial caseous lymphadenitis by age, considering ventilation in sheepfold and control of abscess cases. Error bars indicate 95% confidence intervals. Lambs: <6 months, Young: 6 to 24 months, Adult: > 24 months. “No control” includes non-regular control (sporadic control); “regular control” includes periodic detection and treatment of abscesses before their spontaneous rupture. Four ventilation levels in barns were categorised: maximum ventilation, in barns without a roof; ample ventilation, in barns with wide bilateral openings; medium ventilation, in barns with wide unilateral openings; and poor ventilation, in barns with reduced unilateral openings.

Following the stepwise selection of significant estimators, the final model comprised animals’ age, ventilation of barns, and the practice of abscess control, all of which were deemed significant variables (*p* < 0.001) (see Table 3). Notably, young sheep exhibited a higher prevalence compared to both lambs and adult sheep. Regarding barn ventilation, CL prevalence was higher in environments characterized by poor ventilation compared to those with maximum, ample, or medium ventilation. In terms of abscess control, animals subject to regular abscess control exhibited a significantly lower prevalence of CL compared to those reared without a regular form of abscess control or those reared without any form of abscess control (Figure 2).

### 3.2. Association between Visceral and Superficial Forms of Caseous Lymphadenitis

The presence of *C. pseudotuberculosis* was confirmed in all clinical cases identified in slaughterhouses through quadruplex-PCR. Among all infected animals, the prevalence of visceral abscesses was notably higher in adult sheep (83%, OR = 9.8, 95% CI = 5.5–17.2) compared to young sheep (35%). In terms of the association between superficial and thoracic abscesses, out of 132 sheep with thoracic abscesses (65 young and 66 adult sheep), only 11% (*n* = 15) were affected by both types of abscesses, with no significant differences observed between young (12%) and adult animals (11%; OR = 0.9, 95% CI = 0.3–2.5, *p* = 0.784). This suggests that approximately 89% of sheep with thoracic abscesses did not exhibit any superficial lesions.

**Figure 3:**
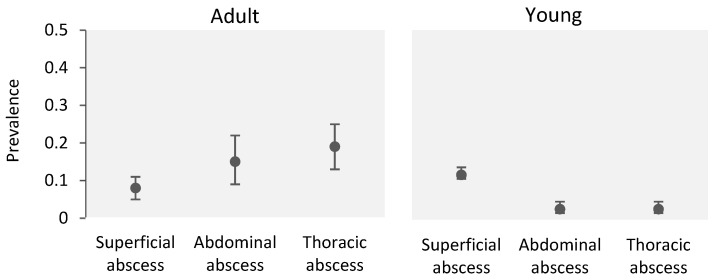
Proportion of adult and young animals with different abscess locations according to animals’ age. Error bars indicate 95% confidence intervals. Superficial abscess: parotid, retropharyngeal, submandibular, prescapular, prefemoral, and popliteal infected lymph nodes and superficial abscesses located in the inguinal, neck, testicle, and scrotum areas; Abdominal abscess: abscesses located in liver and intestines; Thoracic abscess: lung, mediastinal, and bronchial lymph nodes and heart-localized abscesses.

Figure 3 illustrates the prevalence levels of superficial, abdominal, and thoracic abscesses in adult and young sheep. Regarding the association between abscess location and animals’ age in adult sheep, the prevalence of the superficial form was significantly lower (8%, OR = 0.1, 95% CI = 0.06–0.25, *p* = 0.0001) compared to the prevalence of abdominal abscesses (15%). However, no significant difference was observed between the prevalence levels of thoracic (19%, OR = 1.2, 95% CI = 0.6–2.7, *p* = 0.569) and abdominal (15%) abscesses.

**Figure 4:**
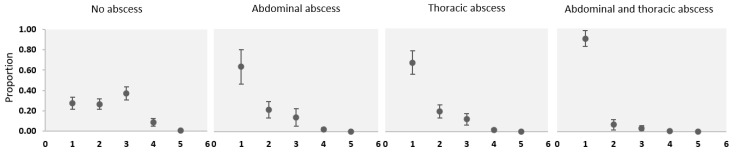
Body score distribution as a function of abscess location in adult sheep. Body scores range from 1 (very thin) to 5 (very fat). Error bars indicate 95% confidence intervals. Abdominal abscess: liver- and intestines-located abscesses; Thoracic abscess: lung, mediastinal, and bronchial lymph nodes and heart-localized abscesses; superficial abscesses have no significant effect (*p* = 0.62; data not displayed).

Additionally, Figure 4 shows a significant association between body score and presence of the visceral form observed in adult sheep. Body score was significantly associated with thoracic (OR = 0.2, 95% CI = 0.1–0.3, *p* = 0.0001) and abdominal abscesses (OR = 0.2, 95% CI = 0.1–0.4, *p* = 0.0001). No significant effect on body score was found in animals with the superficial form of LC (OR = 1.2, 95% CI = 0.6–2.6, *p* = 0.624).

## 4. Discussion

Caseous lymphadenitis (CL) stands as a significant challenge for sheep breeding worldwide, inflicting considerable economic losses. This adverse impact primarily stems from the diminished prices and restricted marketability of sheep with superficial abscesses, alongside the loss of body weight associated with the visceral form. Additional losses emanate from the partial trimming, or the total rejection of carcasses manifesting severe infection and declared unfit for human consumption. In carcasses with minor infection, a removal of the impacted tissues is performed to prevent carcass contamination [4,12]. Our findings underscored that nearly all visited flocks (95%) exhibited clinical cases of superficial CL. This result resonates with the findings of Kichou et al. in the Eastern region of Morocco, where 106 out of 107 flocks were clinically affected [28]. However, the overall individual clinical prevalence of CL (31.1%) observed in our study slightly surpassed the previously reported figure of 28%. Furthermore, the prevalence we uncovered is markedly higher than those documented in sheep from Egypt (19.23% and 3.71%) and Iraq (0.94%) [17,29,30].

Our results pinpointed that the most significant risk factors for the spread of CL were barns with poor ventilation, the absence of abscess control practices, and the age of the animals (with young ones being the most affected among those examined).

Concerning barn conditions, it became evident that poor ventilation was closely linked to the highest CL risk. This association may be attributed to local breeding practices grounded in the belief that reducing barn openings accelerates the fattening process, particularly for animals housed indoors. However, adequate ventilation is a critical requirement in designing a suitable shed [31]. Effective ventilation facilitates proper air circulation, thereby minimizing contamination via aerosols. Additionally, lower survival rates of *C. pseudotuberculosis* have been associated with rapid humidity reduction [32]. It is noteworthy that ample openings in barns allow abundant sunlight entry, exposing bacteria present in the soil to external environmental conditions. Indeed, several studies have demonstrated that decreased biofilm formation by *C. pseudotuberculosis* correlates with exposure to thermal stress and ultraviolet radiation [33,34]. In traditional rearing management, where animals spend most of their day outdoors under the sun, this exposure could provide some level of protection against CL infection.

Another noteworthy factor highlighted in Survey 1 was the efficacy of abscess control in reducing the risk of superficial CL. Flocks where breeders promptly detected and treated abscess cases before spontaneous rupture experienced a decrease in superficial CL incidence. This proactive approach to identifying infected sheep and administering treatment helps prevent naive animals from coming into contact with bacteria-containing pus [35]. Spontaneous abscess ruptures, if not adequately disinfected, pose a significant risk of contaminating other animals. Furthermore, abscess material can contaminate the barn environment, soil, and equipment [36]. Given that *C. pseudotuberculosis* biovar Ovis can survive for extended periods (ranging from 80 to 210 days) in the presence of organic materials, it can infect other sheep through skin lesions [34,37,38]. Deficiencies in preventive hygienic measures for CL, such as a lack of isolation measures for infected animals, have also been reported as risk factors for CL [28].

Regarding the age of sheep within flocks, young animals aged between 6 to 24 months exhibited a high prevalence of superficial CL. Contamination among young sheep is facilitated by the immaturity of their immune systems [4]. Additionally, this category of sheep often undergoes fattening periods during which they are housed indoors for extended durations, typically two to three months, or longer in some cases. Indeed, intensive sheep farming systems, characterized by high density and poor ventilation, have been associated with elevated CL prevalence in countries like the Netherlands and Ireland [39,40]. However, in the Eastern region of Morocco, no discernible difference in CL prevalence related to age categories (young, less than one year; and adult sheep, more than two years) at the farm level was observed due to the repeated exposure of adult sheep to the infection [28]. Other studies have reported that animals aged between 2 and 3 years are at high risk of CL due to wool shearing, which is not practiced in younger animals [21].

Surprisingly, at slaughterhouses, a remarkably high percentage (89%) of animals with thoracic abscesses did not exhibit any superficial lesions, whether in young or adult sheep. This suggests that CL prevalence based solely on clinical signs, as assessed in Survey 1, is likely significantly underestimated. Moreover, adult sheep demonstrated a heightened risk of thoracic and abdominal CL abscesses compared to superficial ones. This could be attributed to a higher culling rate among young animals presenting superficial lesions, thereby retaining animals with healthy skin for reproduction on farms, unaware of the internal lesions. The elevated prevalence of the thoracic form, encompassing lung parenchyma and mediastinal lymph nodes, implies an increased transmission risk through aerosols [35]. When abscesses manifest in mediastinal lymph nodes, sheep may disseminate infectious agents following fistulation to the bronchi, leading to aerosol transmission to other sheep [6,35]. A previous study reported a high prevalence of CL among animals aged more than four years in the Eastern region of Morocco [28]. However, Khanamir et al. observed the highest CL prevalence in sheep and goats older than one year in Iraq [29].

Survey 2 also underscored that thoracic and abdominal abscesses in adult sheep were associated with poor body condition scores. Similarly, body weight loss was linked to the CL-visceral form in infected goats [19,41,42]. This could be attributed to reduced appetite and compromised nutrient digestion and absorption [42]. Furthermore, the visceral form of CL might be associated with chronic wasting syndrome or “thin ewe syndrome” [43]. It has been suggested that the visceral form of CL could either be a significant contributing factor to thin ewe syndrome or a consequence of this syndrome [43]. Moreover, it is recommended to include CL in the differential diagnosis of emaciated ewes [44]. Thus, poor body condition should be factored into CL clinical diagnosis, particularly in adult sheep.

One limitation of the present study was the disparity in the age and sex distribution of the studied sheep samples between flocks and slaughterhouses, due to the differences in the animals undergoing our investigations. Indeed, there was a higher proportion of young sheep and females found in slaughterhouses compared to flocks. Young Sardi females older than six months are primarily designated for slaughter. Conversely, Sardi males are seldom slaughtered for regular meat consumption, as they are reserved for religious celebrations, which explains the limited number of males found in our slaughterhouse sample (*n* = 78).

We encourage the dissemination of the current results as an update for educational programs of future veterinarians and technicians. Additionally, sensitizing workshops about the current findings have been realised for breeders in the studied area, although a wider diffusion is necessary to combat the spread of misinformation. Modern communication tools such as Facebook, Instagram, Twitter, and others could be helpful in sharing the important findings with an expanded audience of veterinarians, technicians, and breeders. A recent study about the usage of Instagram as a social media platform for teaching and engagement in the field of dairy cow nutrition and management showed inspiring outcomes [45]. Given the poor literacy of the rural population, reels and videos may be particularly useful for sheep-breeders’ sensitization to our findings.

## 5. Conclusions

The risk factors implicated in CL within Settat province are closely tied to deficiencies in breeding management rooted in local practices. Key factors include poor barn ventilation, lack of CL case control, absence of quarantine measures, and the age group of 6 to 24 months. Additionally, findings from the slaughterhouse survey indicate that sheep over 24 months exhibit a higher prevalence of thoracic and abdominal CL abscesses compared to superficial ones. A low body score strongly correlates with the presence of visceral forms of CL.

Currently, there is no control program for CL prevention implemented in Settat province, Morocco. Any future program aimed at preventing this disease in sheep, particularly in the studied region, should prioritize the improvement of ventilation in sheep barns and instituting regular control measures for abscess cases, including proper treatment procedures, with a focus on young sheep less than 24 months old. Furthermore, poor body condition should be taken into account in the clinical diagnosis of CL in adult sheep.

## Figures and Tables

**Table 1:** Description of explanatory variables screened in Survey 1.

Explanatory Variable	Description
Sex	Male
	Female
Age group	Lamb (<6 months)
	Young (6 to 24 months)
	Adult (>24 months)
Body score	Score 1 (very thin)
	Score 2
	Score 3
	Score 4
	Score 5 (very fat)
Rearing system applied during 2–3 months preceding flock investigation	Intensive
	Semi-extensive
Flock size	Quantitative variable
Density	Number of heads per square meter
Ventilation level in the building	Level 1: maximum ventilation in barns without roof
	Level 2: ample ventilation in barns with wide bilateral openings
	Level 3: moderate ventilation in barns with wide unilateral openings
	Level 4: poor ventilation in barns with reduced unilateral openings
Presence of traumatizing feeders	No
	Yes
Presence of traumatizing drinkers	No
	Yes
Presence of traumatizing fences	No
	Yes
Presence of thorny plants in the barn’s building	No
	Yes
Frequency of cleaning of the barn	Twice a week
	Weekly
	Daily
Frequency of disinfecting of the barn	Monthly
	Twice a year
	Annually
	Never
Product used for disinfecting livestock buildings	Aldehyde
	Lime
	Lime, Aldehyde
	Bleach
	Bleach, Lime
	Bleach, Aldehyde
	Oil of cade
	Nothing
Crawlspace	No
	Yes (sometimes)
Control of abscess cases: detection and treatment of cases before their spontaneous rupture	Non-control of abscesses
	Non-regular control of abscesses: sporadic detection and treatment of cases before their spontaneous rupture
	Regular control of abscesses: periodic detection and treatment of cases before their spontaneous rupture
Treatment of abscess cases	Bleach
	Bleach + antibiotic
	Oil of cade
	Non-treatment
Isolation of CL cases	No
	Yes
Destruction of pus	No
	Yes

**Table 2:** Univariable analysis models with variables collected in Survey 1, carried out at the farm level.

Explanatory Variable	Examined Animals	CL Cases	OR (95% CI)	*p* Value
**Sex**				**0.003**
Male	1062	260	2.3 (1.3–3.6)	0.003
Female	459	276	1	
**Age group**				**0.001**
Lamb (<6 months)	318	69	1	
Young (6 to 24 months)	572	292	3.8	0.001
Adult (>24 months)	631	114	0.8	0.561
**Body score (from 1 to 5)**				**0.002**
Score 1	170	33	1	
Score 2	359	86	1.3 (0.8–2.12)	0.272
Score 3	566	200	2.3 (1.3–3.8)	0.003
Score 4	341	109	1.9 (1.1–3.3)	0.015
Score 5	85	86	5.1 (1.9–13.9)	0.002
**Lifestyle in last 2 or 3 months**				**<0.0001**
Intensive	384	240	1	
Semi-extensive	1137	235	0.15 (0.1–0.3)	<0.0001
**Flock size**				**0.97**
Continuous variable	1521		0.99 (0.99–1.00)	0.97
**Density (Nb of heads/square meter)**				**0.022**
Continuous variable	1521		1.7 (1.1–2.6)	0.022
**Ventilation level in building**				**<0.0001**
Level 1 (maximum ventilation) ^1^	324	48	1	
Level 2 (ample ventilation) ^2^	719	166	1.7 (1.2–2.45)	0.002
Level 3 (medium ventilation) ^3^	252	91	3.25 (2.2–4.8)	<0.0001
Level 4 (poor ventilation) ^4^	226	170	17.4 (11.3–26.8)	<0.0001
**Traumatizing feeders**				**0.765**
No	1011	323	1	
Yes	510	152	0.9 (0.5–1.8)	0.765
**Traumatizing drinkers**				**0.06**
No	1152	403	1	
Yes	369	72	0.45 (0.2–1.03)	0.06
**Traumatizing fences**				**0.028**
No	618	253	1	
Yes	903	222	0.5 (0.2–0.9)	0.028
**Presence of thorny plants in barn’s building**				**0.099**
No	827	304	1	
Yes	694	171	0.6 (0.3–1.1)	0.099
**Frequency of cleaning barns**				**0.011**
Twice a week	95	35	1	
Weekly	480	258	2 (0.96–4.1)	0.062
Daily	946	182	0.4 (0.2–0.8)	0.011
**Frequency of disinfecting barns**				**0.001**
Monthly	39	3	0.7 (0.1–4.9)	0.761
Twice a year	60	6	1	
Annually	493	123	3.0 (1.1–8.1)	0.032
Never	929	343	5.3 (1.9–14.1)	0.001
**Product used for disinfecting barns**				**<0.0001**
Aldehyde	46	7	1	
Lime	270	59	1.5 (0.6–3.9)	0.342
Lime, Aldehyde	21	0	_	
Bleach	70	22	2.5 (1.2–5.6)	0.019
Bleach, Lime	107	26	1.8 (0.9–3.6)	0.105
Bleach, Aldehyde	17	9	6.3 (4.2–9.2)	<0.0001
Oil of cade	61	9	0.96 (0.5–1.9)	0.918
Nothing	929	343	3.3 (1.8–5.9)	<0.0001
**Crawlspace**				
No	1500	475	_	
Yes (sometimes)	21	0	_	
**Control of abscess cases**				**<0.0001**
Non-control of abscess	380	164	1	
Non-regular control of abscess ^5^	927	297	0.6 (0.3–1.2)	0.166
Regular control of abscess ^6^	214	14	0.1 (0.4–0.2)	<0.0001
**Treatment of abscess cases**				**0.03**
Bleach	888	226	1	
Bleach + antibiotic	138	36	1.03 (0.4–2.8)	0.0948
Oil of cade	115	49	2.2 (0.5–9.1)	0.281
Non treatment	380	164	2.2 (1.1–4.6)	0.03
**Isolation of CL cases**				**0.016**
No	1482	470	1	
Yes	39	5	0.3 (0.12–0.8)	0.016
**Destruction of pus**				**0.018**
No	1361	453	1	
Yes	160	22	0.3 (0.1–0.8)	0.018

^1^ Level 1: maximum ventilation in barns without roof; ^2^ Level 2: ample ventilation in barns with wide bilateral openings; ^3^ Level 3: medium ventilation in barns with wide unilateral openings; ^4^ Level 4: poor ventilation in barns with reduced unilateral openings; ^5^ Non-regular control of abscess: sporadic detection and treatment of cases before their spontaneous rupture; ^6^ Regular control of abscess: periodic detection and treatment of abscess before their spontaneous rupture. CL: Caseous lymphadenitis; OR: Odds ratio; CI: Confidence interval. The bold indicate the analysed explanatory factors and their univariable model *p*-values.

**Table 3:** Final model of multivariable analysis of risk factors of CL in animals investigated at the flock level.

Explanatory Variable	Examined Animals	CL Cases	Prevalence %	OR (95% CI)
**Age group**			0.002	
Lamb (<6 months)	318	69	21.6	1
Young (6 to 24 months)	572	292	51.0	3 (1.54–5.7)
Adult (>24 months)	631	114	18.1	1.1 (0.6–2.2)
**Ventilation level in building**			<0.0001	
Level 1 (maximum ventilation) ^1^	324	48	14.8	1
Level 2 (ample ventilation) ^2^	719	166	23.1	1.3 (0.8–2.16)
Level 3 (medium ventilation) ^3^	252	91	36.1	1.9 (1.1–3.3)
Level 4 (poor ventilation) ^4^	226	170	75.2	8.5 (5.1–14.1)
**Control of abscess cases**			<0.0001	
Non-control/non-regular control ^5^	1307	461	43.1	1
Regular control ^6^	214	14	27.2	0.2 (0.1–0.46)

^1^ Level 1: maximum ventilation in barns without roof; ^2^ Level 2: ample ventilation in barns with wide bilateral openings; ^3^ Level 3: medium ventilation in barns with wide unilateral openings; ^4^ Level 4: poor ventilation in barns with reduced unilateral openings; ^5^ Non-regular control of abscess: sporadic detection and treatment of cases before their spontaneous rupture; ^6^ Regular control of abscess: periodic detection and treatment of abscess before their spontaneous rupture. CL: Caseous lymphadenitis, OR: Odds ratio, CI: Confidence interval.

## Data Availability

The original contributions presented in the study are included in the article, further inquiries can be directed to the corresponding author/s.
